# Japanese encephalitis: Current status, recent progress, and persistent challenges

**DOI:** 10.1371/journal.pntd.0014641

**Published:** 2026-08-18

**Authors:** Susan L. Hills, Huong Minh Vu, Punnee Pitisuttithum, Chang-Kweng Lim, Christin H. Goodman, Joachim Hombach, Tom Solomon

**Affiliations:** 1 Arboviral Diseases Branch, Division of Vector-Borne Diseases, US Centers for Disease Control and Prevention, Fort Collins, Colorado, United States of America; 2 Independent Expert, Ha Noi, Viet Nam; 3 Department of Clinical Tropical Medicine, Faculty of Tropical Medicine, Mahidol University, Bangkok, Thailand; 4 Laboratory of Arboviruses, Department of Virology 1, National Institute of Infectious Diseases, Japan Institute of Health Security, Tokyo, Japan; 5 Independent Expert, Geneva, Switzerland; 6 The Pandemic Institute and the National Institute for Health and Care Research (NIHR) Health Protection Research Unit in Emerging and Zoonotic Infections, University of Liverpool, Liverpool, United Kingdom; 7 Walton Centre NHS Foundation Trust (a Member of Liverpool Health Partners), Liverpool, United Kingdom; Institute of Continuing Medical Education of Ioannina, GREECE

## Abstract

Japanese encephalitis (JE), a mosquito‑borne, vaccine‑preventable disease, is endemic to much of Asia and parts of Oceania. Its main clinical manifestation, encephalitis, is associated with a 20–30% case-fatality rate and long-term sequelae in 30%–50% of survivors. During the past two decades, substantial progress has been made in JE prevention and control. Notably, several countries have introduced JE vaccine into their national immunization programs, leading to marked reductions in morbidity and mortality. Nonetheless, major challenges remain, particularly because the disease cannot be eradicated meaning high-quality, long-term JE vaccination programs must be maintained. In addition, JEV has emerged in an increasingly wide geographic area, including in widespread parts of Australia, and shifts in the age distribution of cases towards adults in some JE-endemic areas have occurred. In this review, we summarize current knowledge on JE, outline recent advances in prevention and control, and describe key persisting and emerging concerns. Addressing these issues and continuing efforts to strengthen JE immunization programs will be important to reducing the burden of this disease for populations in endemic areas now and in the future.

## Author summary

Scientists first isolated Japanese encephalitis (JE) virus, which can cause severe and often fatal disease in humans, in 1935. Subsequently, researchers showed that the virus circulates in nature in a transmission cycle involving mosquitoes, pigs, and wading birds and that humans are not part of the main transmission chain and are only incidentally infected. Over time, countries across much of Asia and parts of Oceania recognized JE as an important cause of encephalitis. Vaccination programs in countries where JE is common have greatly reduced illness and deaths. Despite this progress, major challenges remain. JE cannot be eradicated, so high-quality immunization programs must be maintained, and the virus is appearing in new areas. In this review, we explore the epidemiology, clinical presentation, diagnosis, management, and prevention of JE, summarize recent advances in JE control, and describe key ongoing and emerging concerns.

## Introduction

Japanese encephalitis (JE), a mosquito-borne, vaccine-preventable disease, remains an important cause of death and long-term disability in Asia and parts of Oceania. Because JE is endemic only in this geographic region, the disease has received comparatively little global attention. Although periodic large outbreaks attract short-term interest, awareness of the public health burden of JE, as well as the substantial advances in its prevention and control, remains limited [1]. In this review, we summarize current knowledge of JE virus (JEV) and JE disease, including epidemiology, transmission dynamics, clinical presentation, and prevention strategies, and discuss progress made and remaining challenges in prevention and control.

## History

### Identification of JEV and its enzootic transmission cycle

As early as 1871, seasonal encephalitis outbreaks consistent with JE occurred in Japan, but the first major epidemic, resulting in 6,125 reported cases, occurred in 1924 [2,3]. In 1933, researchers inoculated monkeys with brain emulsions from five individuals from western Japan with fatal encephalitis and the animals subsequently developed neurologic disease [4]. In 1935, JEV was first isolated from patients who died of encephalitis; one isolate, designated the Nakayama strain after the patient’s surname, became the prototype JEV strain and was used in early vaccine development [5,6]. The role of mosquitoes in JEV transmission was inferred from outbreak investigations in Japan and confirmed by the isolation of JEV from *Culex tritaeniorhynchus* in 1938 [7]. Subsequent field studies delineated JEV’s enzootic cycle involving pigs and wading birds [8].

### Early recognition and regional expansion of JEV

During the first half of the 20th century, JE was recognized principally in temperate areas of Asia with regular outbreaks in Japan, Korea, and China [9,10]. Elsewhere, recognition of its public health importance emerged more gradually. In South Asia, India provided the first definitive evidence of JEV transmission through population serosurveys (1952), confirmed clinical cases (1955), and virus isolation from the brain of a fatal case (1958) [11]. During the next 10–20 years, JE was documented in additional South Asian countries, ultimately recorded as far west as Pakistan’s Indus Valley. In Southeast Asia, sporadic viral encephalitis cases were reported from the early 1900s, but awareness rose after a large 1969 epidemic in the Chiang Mai Valley, Thailand [12]. Outbreaks in northern Vietnam from the late 1960s and transmission recognized elsewhere in Southeast Asia during the 1970s led to broader recognition of JE’s regional importance [13].

In the western Pacific region, outbreaks were recorded on Guam (1947–1948) and Saipan (1990), and serological evidence from 1989 indicates JEV’s presence in Papua New Guinea [14–16]. During the 1990s, southward expansion occurred with human cases detected in northern Australia, initially in the outer Torres Strait Islands (1995), and then on the far north Australian mainland (1998). From 2021, more widespread transmission of JEV in Australia was recognized [17].

## Description of the pathogen

JEV is in the *Orthoflavivirus* genus of the *Flaviviridae* family. The JE serocomplex includes Murray Valley, West Nile, Kunjin, Usutu, and other viruses. The JEV genome is a single-stranded, positive-sense RNA molecule approximately 11kb long. It encodes a polyprotein precursor that is cleaved into three structural—capsid, precursor pre-membrane/membrane, and envelope—and seven nonstructural proteins.

JEV is divided into five genotypes that likely arose from a common ancestral virus in the Indonesia-Malaysia region [18]. Historically, genotype (G) III was dominant across most of Asia until GI began to displace it in many countries in the 1990s [19]. GI and, to a lesser extent, GIII remain the main circulating genotypes, but recent isolations of GIV in Australia and GV in Korea and China have raised concerns about their potential reemergence [20]. Although JEV strains comprise a single serogroup, antigenic variation is evident between and within genotypes [21,22].

## JEV transmission

**Fig 1:**
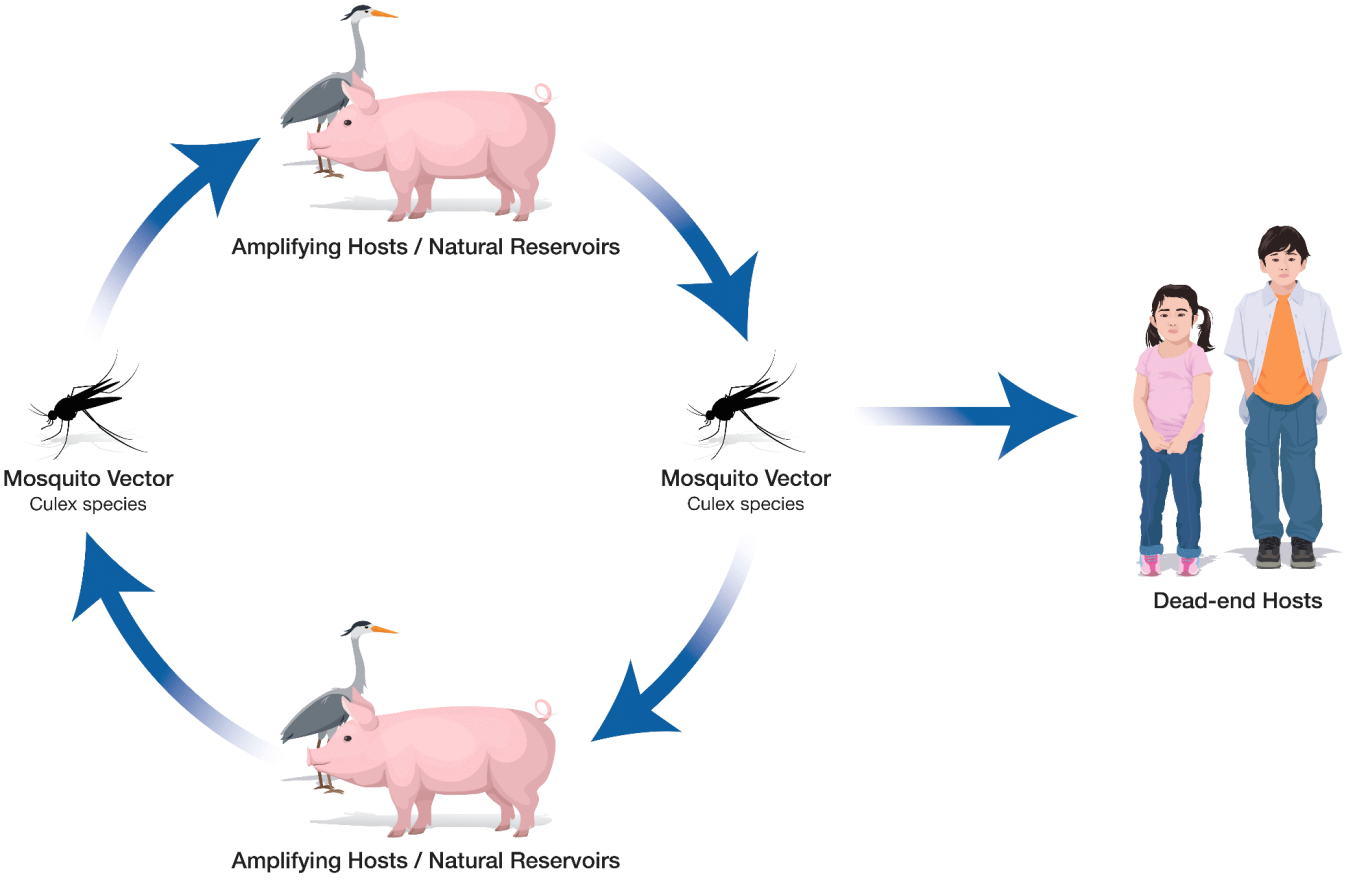
Transmission cycle of Japanese encephalitis virus.

JEV is maintained in an enzootic cycle involving mosquitoes and vertebrate hosts, primarily pigs as the main amplifying hosts and wading birds in the Ardeidae family (e.g., herons, egrets) as maintenance hosts (Fig 1). Infected mosquitoes transmit JEV to humans, who are considered dead-end hosts because of brief and low-level viremia which is of insufficient magnitude to infect mosquitoes [23].

*Culex* mosquitoes, especially *Cx. tritaeniorhynchus*, are the main vectors of JEV in most endemic locations. *Cx. tritaeniorhynchus* is an evening- and nighttime-biting mosquito, is mainly exophagic, and shows a feeding preference for large domestic animals and birds over humans [24]. Larval habitats include flooded rice paddies, marshes, and other stagnant collections of water. Rice fields, in particular, can yield extremely high mosquito numbers, with surveys estimating up to 30,000 adult mosquitoes produced daily from an average-sized field [25]. Although JEV has been isolated from more than 30 mosquito species, not all are competent vectors; studies suggest at least 17 are confirmed vectors and 10 are potential vectors [26]. Although JEV RNA has been detected in midges and ticks, they are unlikely to play any important role in transmission [27,28].

The role of domestic pigs as the principal amplifying hosts of JEV stems from their high-titer viremias after infection and the regular introduction of susceptible offspring related to rapid turnover in populations. Pig infections are usually asymptomatic, although infections in pregnant sows can result in reproductive problems (i.e., aborted or mummified fetuses, stillborn or weak piglets), and in boars can lead to orchitis and reduced fertility [29]. JEV transmission between pigs is mainly by mosquitoes. Direct transmission through oronasal secretions has been documented, but it is not known whether this route has any role in sustaining virus circulation [30]. Pigs are the main amplifying hosts in the enzootic cycle, but JEV outbreaks and transmission in locations where pig populations are minimal or absent (e.g., in areas in Malaysia, Indonesia, and Bangladesh) indicate transmission can be sustained without their involvement [31–33]. Wading birds typically experience asymptomatic infections and function as maintenance (reservoir) hosts. Viremias in ducklings, chicks, bats, and sheep are likely high enough to infect mosquitoes, but the contribution of these animals to JEV transmission is unclear [34–36]. Horses are well recognized as being susceptible to neurologic disease, other species (e.g., seals, meerkats, and cows) have occasionally been reported with encephalitis, and serologic evidence of infection has been found in many other animals (e.g., dogs, goats, rodents, water buffalo); however, none of these animals are believed to contribute to transmission [37].

Although almost all JEV transmission among humans occurs via mosquito bites, less common routes have been identified. Transplacental transmission was confirmed when four miscarriages occurred among infected pregnant women during outbreaks in India and JEV was isolated from one of the aborted fetuses [38,39]. Transmission through blood transfusion and probable transmission through liver transplantation have also been described [40–42]. Finally, at least 22 laboratory-acquired JEV infections have been reported [43].

## Epidemiology

### Geographic distribution and incidence

**Fig 2:**
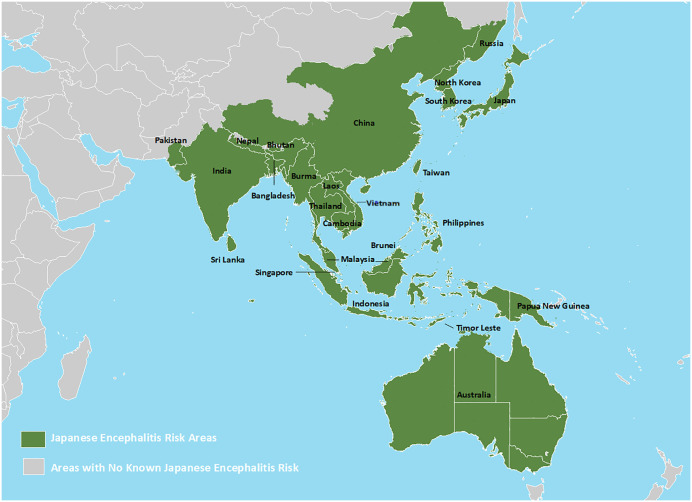
Japanese encephalitis virus risk area in Asia and Oceania.

JE occurs throughout most of Asia and parts of the western Pacific, a region that accounts for over 50% of the world’s population (Fig 2). JE is the leading recognized cause of encephalitis in most of the 24 countries in this geographic risk area [44]. Each year, roughly a few thousand cases are reported to the World Health Organization (WHO), but this number underestimates true disease burden because of the limited surveillance and diagnostic capacity in many countries [45]. A 2011 systematic review that included only high-quality, population-based studies with laboratory-confirmed cases estimated that 67,900 JE cases typically occur annually, though some modeling studies have produced higher estimates [46–48]. The systematic review estimated about 13,600–20,400 deaths and 14,300–27,200 cases with long-term sequelae occur annually. The overall disease incidence in endemic areas was 1.8 cases per 100,000 population, and for children aged <15 years was 5.4 cases per 100,000. Incidence can vary considerably by year and location, and outbreaks can result in thousands of cases in a single year.

JEV has increased its range in recent decades, including to higher altitudes in Nepal [49]. The most dramatic recent geographic expansion was seen in Australia. Following recognition in the 1990s of JE cases in the outer Torres Strait Islands (between the Australian mainland and Papua New Guinea; 3 in 1995 and 1 in 1998) and the far northern mainland (Cape York; 1 in 1998), no locally acquired cases were reported for more than two decades, until two cases with infection acquired in the Northern Territory (central-northern region of Australia) occurred during the first half of 2021 [17,50,51]. JEV activity was subsequently documented in extensive areas of mainland Australia and more than 50 confirmed and probable cases have been reported through May 2026 [52].

A small number of reports have described probable JEV presence outside the Asia-Pacific region. In 2023, a probable JEV infection likely acquired in a seaport area of southern California, USA, resulted in JEV transmission to a liver transplant recipient [41]. During a 2016 yellow fever outbreak in Angola, high-throughput sequencing of serum from a patient clinically diagnosed with yellow fever indicated co-infection with JEV and yellow fever virus [53]. In Italy, RNA fragments consistent with the JEV genome were identified in seven dead birds and the bone marrow of four apparently healthy birds collected between 1997 and 2000, as well as in a pool of *Culex pipiens* mosquitoes collected in 2010 [54]. The epidemiological significance of these reports, in the absence of evidence of sustained transmission, remains uncertain.

### Age-related and seasonal disease patterns

**Fig 3:**
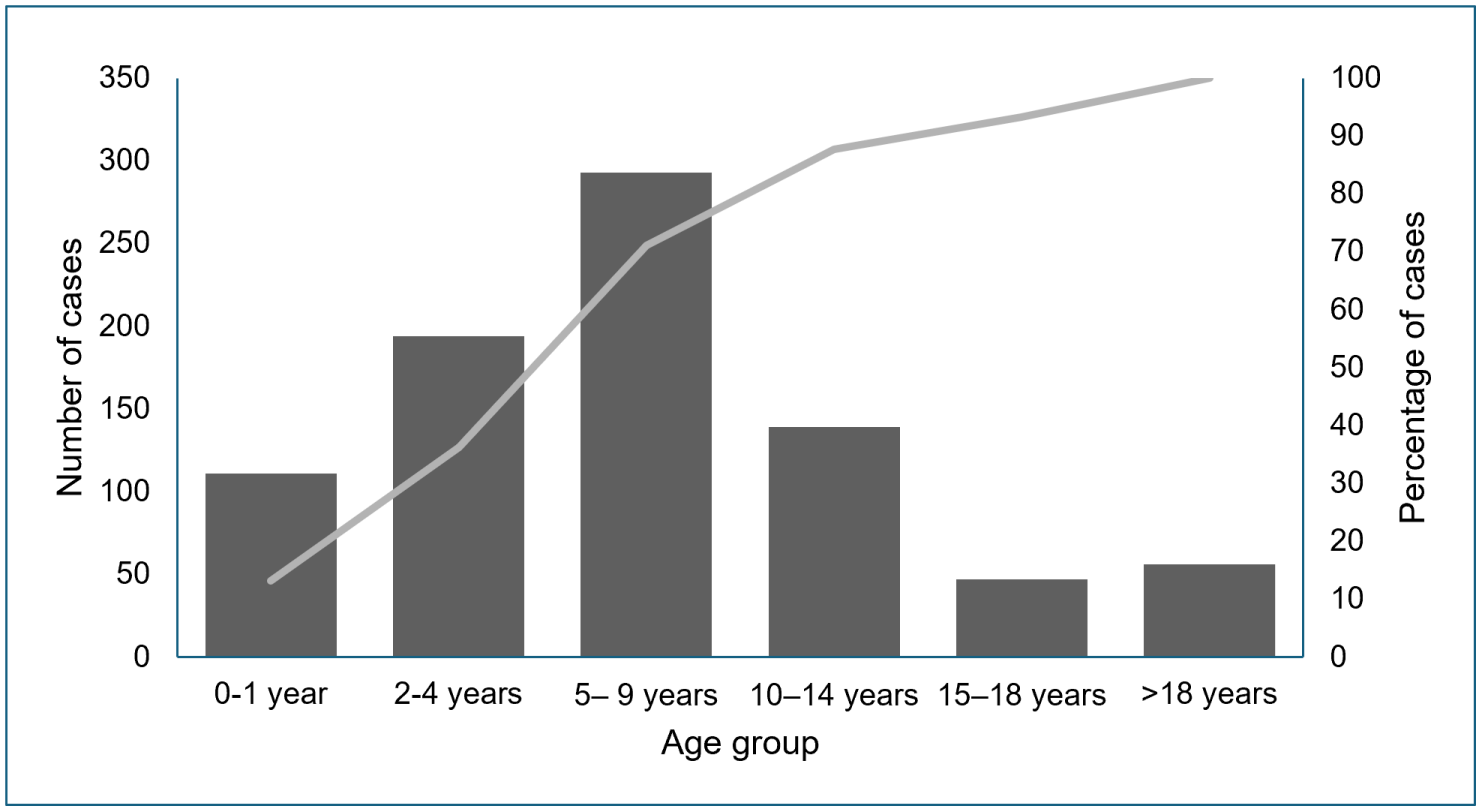
Confirmed Japanese encephalitis cases by age group in the Philippines, 2014–2017, showing typical predominance in children aged <15 years. *Data source: Lopez AL, Raguindin PF, Aldaba JG, Avelino F, Sy AK, Heffelfinger JD, et al. Epidemiology of Japanese encephalitis in the Philippines prior to routine immunization. Int J Infect Dis. 2021;102:344-51*.

In most endemic areas, JE primarily affects children, with the vast majority of cases occurring among individuals aged <15 years; most adults have acquired protective immunity through natural exposure to the virus (Fig 3) [55]. In some settings where routine JE immunization has prevented childhood cases—for example, Japan, Korea, Taiwan, and parts of India and China (principally northern China)—the age distribution of cases has shifted toward adults, particularly the elderly [56]. Sometimes this shift represents an increase in the proportion of cases among adults without an absolute rise in adult incidence; however, in some instances there has been a true increase in adult cases [57]. Factors likely contributing to cases among older persons include waning vaccine-induced immunity, immunosenescence, and reduced opportunities for natural exposure and immunologic boosting in urban populations. Because travelers from non-endemic countries are usually immunologically naïve, travel-associated JE can occur at any age.

There are two principal patterns of JEV transmission. In tropical areas (e.g., Indonesia, southern Vietnam) transmission persists year-round, commonly intensifying during the rainy season. In temperate areas (e.g., Japan, northern China) transmission is seasonal, with highest case numbers in summer or autumn; however, the season duration and peak transmission months vary by region. In these areas, JEV probably persists from year-to-year by over-wintering and vertical transmission in mosquitoes or possibly by persistence in tissues of hibernating animals such as bats or reptiles [58,59]. Annual reintroduction by viremic migratory birds or windblown mosquitoes might also occur [60,61].

### JE in residents of endemic areas and travelers

JEV transmission primarily occurs in rural agricultural areas, where rice paddies and other wetlands create abundant mosquito breeding habitats. These standing water bodies also attract wading birds. Moreover, in rural settings pigs are often housed close to dwellings. Together the presence of animal hosts and mosquito vectors efficiently sustains the JEV enzootic transmission cycle and creates a high-risk environment for the local population. Residents in rural areas frequently have limited economic resources and JE-related medical and other costs can place severe financial strain on affected families [62,63]. While the highest risk for JE is in rural settings, environments that enable enzootic JEV transmission are sometimes present on the outskirts of Asian cities and JE cases have very occasionally been reported in suburban locations of large metropolitan areas [61,64,65].

JE is also a concern for travelers to JE risk areas. Prior to 1973, >300 cases of JE were reported among military personnel from Australia, Russia, the United Kingdom, and the United States [66]. During 1973–2025, at least 101 JE cases among travelers or expatriates from non-endemic countries who had traveled to JE risk areas were reported in the literature or to the U.S. Centers for Disease Control and Prevention (CDC), although reported cases likely underestimate true numbers [67]. Of 101 reported cases, 60 (59%) occurred in tourists, 17 (17%) in expatriates, 6 (6%) in military personnel, 2 (2%) in individuals on work-related trips, and 16 (16%) in people with unspecified travel type. The work-related cases involved a male who taught in Taiwan for four months and a female who was working for several months as an academic botanist in rural China [66,68]. No cases were reported among business or other shorter-term travelers who visited only urban areas.

## Pathophysiology

JEV pathogenesis is driven by the virus and the host immune response, which can have both protective and pathogenic effects. Following intradermal inoculation of viral particles from a mosquito bite, the virus replicates in dendritic or other skin cells which migrate to draining lymph nodes where additional replication occurs. Spread to the bloodstream and subsequently to the central nervous system (CNS) follows. The mechanism of neuroinvasion is not well understood and multiple possible mechanisms have been proposed (e.g., direct infection of the vascular endothelium, passage within infected cells such as monocytes in a “Trojan Horse” mechanism, retrograde neuronal transport) [69]. On entry, the virus primarily infects neurons.

Infection initially activates an innate immune response, including production of proinflammatory cytokines and type 1 interferons, which help limit viral replication and spread; however, strong inflammatory responses can also contribute to neuropathology. Activation of the adaptive immune response, including humoral (B cell) and T-cell-mediated responses, also occurs. B cells produce virus-specific neutralizing antibodies that limit viremia in the pre-neuroinvasive phase and prevent viral spread to the CNS. CD4+ T helper cells support antibody production and CD8+ cytotoxic T cells recognize and kill infected cells in the brain, although T cells can also cause immune-mediated damage in the brain [70]. Together, the B- and T-cell responses generate immunological memory that can protect against reinfection.

## Clinical manifestations

Most JEV infections are asymptomatic with neurologic disease occurring in fewer than 1% of cases [3]. Among patients who develop clinical disease, the incubation period following a mosquito bite ranges from 5 to 15 days. Acute encephalitis is the main clinical manifestation of illness, although less severe presentations such as aseptic meningitis or febrile illness also occur [71]. Initial symptoms are usually non-specific and can include fever, diarrhea, and rigors followed by headache, vomiting, and generalized weakness. Over subsequent days, mental status changes, focal neurologic deficits (e.g., paresis, hemiplegia, tetraplegia, or cranial nerve palsies), and/or movement disorders develop, and many affected individuals progress to coma. Seizures, usually generalized tonic-clonic, are common among pediatric patients but less frequent in adults [72,73]. In some children, they can manifest as twitching of a digit, ocular deviation, or irregular breathing [74]. Such subtle seizure activity can easily be overlooked on clinical examination and might only be detected through electroencephalographic monitoring. In some patients, the first manifestation can be abnormal behavior or acute psychosis; historical accounts from the Korean War in the 1950s indicate that some American soldiers later confirmed to have JE were initially misdiagnosed as having “war neurosis” [75,76]. A notable clinical presentation of JE is a Parkinsonian syndrome resulting from extrapyramidal involvement; findings include an expressionless facial appearance with wide unblinking eyes, tremor, and cogwheel rigidity [71].

Poliomyelitis-like acute flaccid paralysis caused by JEV infection of anterior horn cells of the spinal cord can also occur. Patients typically have a short febrile illness and then present with rapid onset of paralysis in one or more limbs, usually asymmetric and more common in the lower than upper limbs. Absence of reflexes and lack of sensory symptoms are characteristic. Initially there is no alteration in consciousness, but encephalitis subsequently develops in about 30% of patients [71]. Other uncommon manifestations of JEV infection include acute transverse myelitis, Guillain-Barré syndrome, and probably chorioretinitis and acute disseminated encephalomyelitis [77–80]. In rare cases, an early relapse or secondary phase within 1–2 months of the initial JE illness has been reported as a result of autoimmune encephalitis, especially with anti-N-methyl-D-aspartate receptor (anti-NMDAR) antibodies [81]. The differential diagnosis of JE includes other CNS infections, para- or postinfectious causes, and noninfectious diseases.

### Imaging and electoencephalograhy

Magnetic resonance imaging (MRI), especially T2-weighted and fluid-attenuated inversion recovery (FLAIR) sequences, is the best approach for detecting focal abnormalities [82–84]. Thalamic lesions are the most common abnormality and are typically bilateral [82]. In a systematic review of 181 patients with MRI results, focal lesions were reported in the thalamus in 74% of cases, the basal ganglia in 43%, and the brainstem in 33%; lesions were less frequent in the medial temporal lobe (18%) and cerebral cortex (12%) [84]. In some patients with anti-NMDAR encephalitis, MRI scans demonstrate new or enlarged lesions not evident on imaging obtained during the acute illness [85]. Overall, imaging lacks the specificity to establish a definitive diagnosis; for example, thalamic lesions are frequently also seen in dengue encephalitis [84]. Electroencephalography in JE patients is often non-specific with findings such as theta and delta coma, burst suppression, and epileptiform activity [71].

### Outcome

Reported JE case-fatality rates vary but are generally about 20–30%; some deaths occur after a brief fulminant illness while others follow a more protracted illness course. Among survivors, long-term sequelae occur in at least 30%–50% [3]. The principal sequelae are physical, intellectual, and behavioral and can include recurrent seizures, motor weakness or paralysis, abnormalities of tone and coordination, emotional lability, and memory, cognitive, or speech problems. Subtle deficits (e.g., learning or behavioral problems) may go unrecognized in children who have a good physical recovery. The Liverpool Outcome Score was developed as a simple, reliable tool for use in resource-limited settings to measure disability among children after JE, combining observations of simple motor tasks with a caregiver’s assessment of a child’s ability on several cognitive and functional items compared with peers in their community (e.g., communication skills, recognition of people, performance at school or in routine activities at home) [86]. Using this tool, disability rates of 38–95% within 2.5 years of illness were reported in studies in several Asian countries (i.e., Cambodia, India, Indonesia, Nepal) [62,87–90]. Over time, many children and adults show clinical improvement or resolution of sequelae, but some experience worsening or new sequelae (e.g., seizures, optic nerve degeneration) [91,92]. The case-fatality rate is generally lower in children than in adults, but among survivors more frequent sequelae are reported among children [56].

## Diagnosis

JE cannot be differentiated clinically from most other causes of encephalitis and diagnostic testing is important to confirm infection. The diagnosis of JE is primarily serologic, using an enzyme-linked immunosorbent assay (ELISA) to detect JEV-specific immunoglobulin M (IgM) antibodies in cerebrospinal fluid (CSF) or serum. Because IgM antibodies do not cross an intact blood-brain barrier, the presence of JEV IgM in a non-bloody CSF sample usually indicates a recent CNS infection. Detection of IgM antibody in serum suggests JE but can reflect asymptomatic infection or recent vaccination; one study found that >40% of children vaccinated with the live attenuated SA14-14-2 JE vaccine had serum JE IgM antibodies at 1 month post-vaccination, although IgM antibody was rarely detectable by 6 months [93]. On hospital admission, JEV IgM antibodies are detectable in CSF of 70–90% and in serum in 60%–70% of patients [94,95]. Most patients have IgM antibodies in CSF by 5–8 days after symptom onset and in serum by about 9 days after onset, so if results of the initial IgM testing are negative, a convalescent serum specimen should be obtained. IgM is typically detectable in serum for 2–3 months and can persist for at least 1 year [96]. Within the appropriate clinical and epidemiologic context, a positive IgM test has good diagnostic predictive value, although cross-reactivity with other flaviviruses (e.g., dengue, West Nile, or Murray Valley encephalitis viruses) can occur. Plaque reduction neutralization tests (PRNTs) can be used to discriminate between cross-reacting antibodies or confirm infection if neutralizing antibody titers are at least 4-fold higher for JEV than those for other flaviviruses tested, or there is at least a 4-fold change in titers between acute and convalescent sera. PRNTs are particularly useful in primary arboviral infections but prior flavivirus infection or vaccination complicates diagnosis because of cross-reactive antibodies and original antigenic sin (i.e., the immune system’s tendency to boost antibodies against the original flavivirus after infection with a new flavivirus). Routine access to PRNTs is limited in many countries in Asia except through the WHO JE laboratory network [97].

Virus isolation or detection of ribonucleic acid (RNA) with a nucleic acid amplification test in serum or CSF can provide definitive evidence of infection, but these approaches are insensitive in routine practice because viremia in immunocompetent individuals is low-level and has usually resolved by the time the patient presents to care. Rare reports describe the use of metagenomic next-generation sequencing for diagnosis, or recovering virus or RNA from urine or throat swabs [41,98–100].

## Treatment

No specific therapies have proven effective for JE, but supportive treatment can substantially reduce morbidity and mortality. Early and high-quality clinical management is essential. In a study in Nepal, improved outcome was associated with a shorter interval between illness onset and hospitalization [101]. Therefore, in areas with ongoing JEV transmission, ensuring community awareness of JE’s clinical features and the importance of early presentation to a healthcare facility is vital, particularly in settings where patients might first seek care from traditional healers [101,102]. In health facilities with limited capacity, rapid recognition of a seriously ill patient, clinical stabilization, and prompt referral to a higher-level facility are critical. During hospitalization, patients with encephalitis require close monitoring for development of raised intracranial pressure, seizures, or inability to protect the airway, and for maintenance of adequate cerebral perfusion pressure. Patients with acute flaccid paralysis should be monitored for symptoms or signs of acute neuromuscular respiratory failure (e.g., development of dysphagia or dysarthria). Preventing secondary complications is important, including aspiration pneumonia in any patient with a reduced gag reflex. This can be accomplished through careful management of ventilation, fluids, and nutritional status, and measures to avoid contractures and pressure sores. Controlled clinical trials have assessed various potential therapeutics that have not shown benefit in improving outcomes, including corticosteroids, interferon alfa-2a, ribavirin, minocycline, and intravenous immunoglobulin [103]. However, many of the studies were underpowered, with fewer than 400 patients with confirmed JE enrolled in clinical trials overall. Research continues into other potential treatments that target the virus or regulate the host immune response [104].

## Prevention

### Prevention in endemic areas

Human vaccination is the most effective means of JE prevention and the WHO recommends that JE vaccine be incorporated into immunization programs in all areas where JE is a public health problem [105]. As of May 2026, among the 24 countries with JEV transmission risk, 14 (58%) had implemented and sustained an immunization program nationally or sub-nationally, and an additional 3 (13%) had determined that the level of risk did not warrant a program. The success of human vaccination programs is clear. A study using surveillance data from multiple JE-endemic locations demonstrated disease reductions of 73%–100% in six economically developed countries or territories with longer-term immunization programs and reductions of 14%–79% among children aged <15 years in six low- and low-middle-income countries with more recent programs [106]. Vaccination can also result in economic and social benefits; one study quantifying averted costs of illness estimated that the overall value of JE vaccination in the period from 2001 to 2020 was 4.2 billion USD [107].

At least seven different types of JE vaccines are manufactured and used in one or more countries globally (Table 1). Three of these vaccines are widely available internationally, including 1) the live attenuated SA14-14-2 vaccine produced by Chengdu Institute of Biological Products (CDIBP), China (trade names: CD.JEVAX, RS.JEV), 2) the inactivated Vero cell culture-derived vaccine produced by Valneva Austria GmbH (trade names: IXIARO, JESPECT; also produced by Biological E. Ltd, India after technology transfer from Valneva [trade name: JEEV]), and 3) the chimeric (live attenuated) vaccine produced by Global Biotech Products Co., Ltd, Thailand (trade names: IMOJEV, THAIJEV). These three vaccines are prequalified by WHO, meaning they meet WHO standards for vaccine quality, safety, and efficacy, and are eligible for procurement by United Nations agencies and Gavi.

**Table 1:** Licensed Japanese encephalitis vaccines.

Vaccine type	Substrate	Viral strain	Manufacturer	Countries where available
Inactivated	Vero cells	SA14-14-2	Valneva Austria GmbH, Austria Biological E., India^1^	Valneva: Australia, Europe, Israel, New Zealand, North America Biological E: India
	Vero cells	Beijing-1	Biken, Japan KM Biologics, Japan	Biken: Japan KM Biologics: Japan, Korea^2^
	Vero cells	Beijing P-3	Liaoning Chengda Biotechnology Co., China	China
	Vero cells	Kolar strain (JEV 821564XY)	Bharat Biotech International, India	India
	Mouse brain	Nakayama	Vabiotech, Vietnam	Vietnam
Live attenuated	Hamster kidney cells	SA14-14-2	Chengdu Institute of Biological Products (CDIBP), China Wuhan Institute of Biological Products (WIBP), China	CDIBP: Cambodia, China, Indonesia, Laos, Myanmar, Nepal, Philippines, South Korea, Sri Lanka, Thailand WIBP: China
Chimeric (live attenuated)	Vero cells	JE SA14-14-2/yellow fever 17D	Global Biotech Products Co., Thailand	Australia, Brunei Darussalam, Cambodia, China (Taiwan), Hong Kong, Indonesia, Malaysia, Myanmar, New Zealand, the Philippines, Singapore, South Korea, Thailand, Vietnam

^1^Manufactured in India following technology transfer from Valneva.

^2^Fill finish of KM Biologics JE vaccine by Boryung Pharmaceutical Co., Korea.

Urbanization and improved socioeconomic conditions have likely played a role in reducing JE incidence in endemic areas, but vaccination has been the single most effective means to control JE [105]. Although vector control using chemical or biological (e.g., larvivorous fish) methods can sometimes reduce transmission, particularly during outbreaks, this approach is resource intensive, typically does not achieve sustained reductions in human disease incidence, and insecticide resistance can develop. Immunizing pigs is similarly problematic, because rapid population turnover makes programs financially and logistically burdensome, the interval for effective immunization in young pigs between disappearance of maternal antibodies and potential infection is narrow, and implementation of pig immunization programs is not feasible in settings where pigs roam freely, as is common in many resource-limited settings [108]. Even if this approach were adopted, enzootic JEV transmission can be sustained without the involvement of pigs [29].

### Prevention in travelers

All travelers visiting JE risk areas should use personal protective measures to prevent mosquito bites, including wearing long-sleeved shirts and pants and using insect repellents. In addition, vaccines are important for some travelers visiting risk areas. Vaccination recommendations vary by country but generally target higher-risk travelers given the overall low risk of JE for travelers (i.e., <1 case per million travelers) [67,109]. Some travelers are at higher risk of infection based on factors that increase their risk of JEV exposure including longer periods of travel, spending time in rural areas, participating in extensive outdoor activities, and staying in accommodations without air conditioning, screens, or bed nets (Table 2) [110]. The US Advisory Committee on Immunization Practices recommends JE vaccination for persons moving to a JE-endemic country to live, longer-term (e.g., ≥1 month) travelers, and frequent travelers to JE-endemic areas and indicates JE vaccine should also be considered for shorter-term (e.g., <1 month) travelers if they have an increased risk of JE based on their planned travel location, season, duration, activities, and accommodations [109]. Resources are available that provide specific information on areas with JE risk, but transmission levels can fluctuate from year-to-year [44,67]. Assessing the extent of JEV infection risk can also be complicated by underreporting of cases in some endemic areas and obscuring of viral activity when there are local immunization programs. Web-based resources and tools are available to support informed decision-making [110,111].

**Table 2:** Factors that increase risk for Japanese encephalitis among travelers^*^.

Factor	Related information
**Travel duration**	Highest incidence of disease has been reported among longer-term travelers Although no specific duration of travel puts a traveler at risk for JE, longer-term travel increases the likelihood that a traveler might be exposed to an infected mosquito Longer-term travel includes cumulative periods in JE-endemic areas; this includes frequent travelers and people living in urban areas who are likely to visit higher-risk rural areas
**Season**	JEV transmission occurs seasonally in some areas and year-round in others Information on expected JE virus transmission by country is available from the US Centers for Disease Control and Prevention website (https://www.cdc.gov/japanese-encephalitis/data-maps/index.html)
**Travel location**	The highest risk occurs from mosquito exposure in rural or agricultural areas Mosquitoes that transmit JEV typically breed in flooded rice fields, marshes, and other stagnant collections of water Some JE cases have been reported among travelers to coastal areas or resorts located in or adjacent to rural or rice-growing areas JE can occur in large, focal outbreaks indicating extensive active JEV transmission in those areas
**Activities**	The mosquitoes that transmit JEV feed most often outdoors, particularly from sunset through sunrise, so the highest risk occurs with outdoor activities during that period Examples of higher risk outdoor activities include camping, hiking, trekking, biking, rafting, fishing, hunting, or farming
**Accommodation**	Accommodations without air conditioning, screens, or bed nets increase risk for mosquito exposure

*Available at: https://www.cdc.gov/japanese-encephalitis/hcp/vaccine/index.html; JEV: Japanese encephalitis virus.

## Progress and challenges

### Progress

Up until 2006, routine JE immunization programs covering most or all at-risk areas were in place in just eight countries or territories (Japan, South Korea, Australia, China [Taiwan], Malaysia, Sri Lanka, Thailand, Vietnam), many of which are high-income countries or territories [106,112]. During the past 20 years, remarkable progress has been made, with six additional countries incorporating JE vaccine into their national immunization programs for at-risk areas (Cambodia, China, India, Laos, Myanmar, Nepal); in addition, Indonesia has implemented a program in its highest risk provinces (Bali, Kalimantan). As a result, a substantial global reduction in disease burden has been achieved. Using data from 16 low- and middle-income JE endemic countries, one study estimated that JE vaccination will prevent 240,000 deaths from 2000 to 2030 [113].

Multiple factors have driven progress. Implementation of surveillance programs, if only in sentinel sites in some countries, and wide adoption of WHO JE surveillance standards has improved understanding and raised awareness of disease burden. A WHO JE laboratory network has strengthened diagnostic capacity through training, technical support, and availability of proficiency and confirmatory testing [97]. The development and licensure of several safe and effective vaccines, together with WHO JE vaccination recommendations and Gavi financing, has enabled several lower-income countries to introduce or expand JE immunization programs. Multiple partners such as the Gates Foundation, PATH, universities, and government institutions have supported progress through funding, evidence generation, technical support, and other efforts.

### Challenges

Despite notable advances in disease prevention and control, important challenges—both persistent and emerging—remain.

1. *Need for sustained high-quality surveillance and vaccination programs as JE cannot be eradicated:* As a zoonotic disease with natural viral reservoirs, JE cannot be eradicated, although elimination of clinical disease in humans is possible through universal vaccination of at-risk populations [105]. Individual protection is essential for disease prevention because there is no herd immunity so sustained high vaccination coverage must be maintained. In association with vaccination, surveillance is required to monitor program effectiveness and to detect potential geographic expansion of JEV that could necessitate extension of vaccination efforts. There must be a reliable supply of, and equitable access to, good quality diagnostic tests. Maintaining vaccination and surveillance will require commitment and considerable resources.
2. *Expansion in the JEV transmission area:* JEV has emerged in an increasingly wide geographic area in recent years. Although factors driving vector-borne disease emergence are complex, several factors might favor further expansion. These include increases in rice cultivation and pig production in many areas (augmenting mosquito habitats and increasing JEV amplifying hosts) and land use-related factors (e.g., clearing of land) [114]. Changes in temperature and precipitation patterns could prolong vector seasonal activity, increase larval habitats and mosquito abundance, and make more locations suitable for transmission. The possibility of spread and establishment of JEV is now of concern well beyond traditional JEV transmission areas, with authorities in the United States and Europe identifying the risk and developing response strategies to guide control measures were JEV to be introduced in those areas [115,116].
3. *Shift in age distribution of cases toward adults in some areas:* JE remains primarily a disease of childhood in most JE-endemic countries and strong pediatric immunization programs remain the foundation of JE control, providing long-lasting protection through to adulthood. Nonetheless, vaccination has contributed to a relative increase in cases in older age groups in some locations, raising the question of the need for adult vaccination programs [117]. Before implementing adult vaccination programs, careful, context-specific evaluation is required including consideration of the age-specific incidence and burden of disease, the public health impact and cost-effectiveness of vaccinating older adults, operational feasibility of adult immunization, and vaccine immunogenicity and safety in older persons [56].
4. *Level of vaccine protection with emergence of GV JEV in some locations:* All licensed vaccines are based on Glll JEV strains. The recent detection of GV, the most antigenically distinct JEV, in parts of China and in Korea, including in at least one vaccinated individual, has raised concern about the level of vaccine-induced protection against GV [118]. Previous studies indicated that Glll-based vaccines elicit cross-protective responses against heterologous JEV genotypes [119]. Current GV-related concerns are mainly based on mouse studies showing lower neutralizing antibody levels against GV or reduced survival rates after GV JEV challenge. In addition, in a small human study (*N* = 19), geometric mean titers were lower against three GV isolates than against a Glll isolate. However, of note, most subjects (84%–95%) in this study had protective neutralizing antibody titers against the three GV isolates [120]. Overall, current evidence is insufficient to conclude whether GIII-based vaccines will or will not provide acceptable protection against GV JEVs and further studies and continued monitoring of vaccine effectiveness in the field will be needed.

## Future prospects

JE remains a major public health concern given its severity, spread, and incidence. Beyond mortality, many survivors suffer considerable long-term disabilities. Substantial progress has been achieved through the rollout of immunization programs. Although challenges remain, JE control across all endemic areas is feasible but will require adequate financial resources and sustained advocacy to keep JE vaccination prioritized at national, regional, and international levels. JE will not be eradicated owing to the virus’ enzootic transmission cycle, so continued investment in surveillance and vaccination efforts is essential to improve and protect the health of populations in JE-endemic areas now and in the future.
